# The Brooklyn Dental Society

**Published:** 1879-08

**Authors:** 


### ARTICLE III.

### *The Brooklyn Dental Society.*

The Brooklyn Dental Society held its regular meeting at the residence of Dr. Fry, on Monday evening, Feb. 10th, 1879. Vice-President Dr. A. N. Chapman in the chair.

#### Incidents of Office Practice.

Dr. Rippier: A patient presented himself some time ago to have a left inferior wisdom tooth extracted. It had caused a great deal of irritation in its eruption, the anterior cusp of the tooth pressing against the posterior side of the second molar. Several unsuccessful attempts were made to extract it. It was finally agreed to remove the second molar. The wisdom tooth was loose and could be moved around in its socket, but it was impossible to get it out from its peculiar position. The second molar was extracted, and it drew the wisdom tooth out with it—they both came together. After being cleansed and examined, the second molar was found in the condition you see it, with quite a cavity in the posterior root. I have not the wisdom tooth. The patient took both teeth away and lost the wisdom tooth.

Dr. Atkinson: Is the cavity from friction or decay?

Dr. Rippier: That I leave the gentlemen to judge. I should judge it was from the pressure of the wisdom tooth on the periosteum, causing disease of the bone.

A highly evolved monkey, about 13 months' old, was brought in by one of my patients. The monkey would not eat and was evidently suffering. His master could not make out what the trouble was. His son happened to look in the mouth and the right upper central was found to be affected by acute periodontitis and there was a good deal of swelling of the gum. The monkey did not like the instruments that lay before him. He looked pretty sharp at me. We had trouble to get his mouth open. His master thought it was better to have the tooth out. I desired to save it, but I was not quite certain whether it was a deciduous tooth or not. How were we going to get at it? I finally took what was once the handle of a mallet, and slipped over it a piece of rubber tubing, just about the size of the back teeth, so that he could bite through it and not hurt his teeth, and extracted the offender. It was as black as you see. It was in quite firm. After he got it out, he seemed greatly relieved. He gave one little yell; his master told him to spit, and he did it as gracefully as any one would. The eye teeth were beautifully clean and the gums healthy and the breath so sweet that it would shame some of our patients.

Dr. Atkinson: How old was he?

Dr. Crandell: Thirteen months.

Dr. Atkinson: I guess this is a deciduous tooth. I am not up on monkeyology.

A voice asked Dr. Atkinson's opinion as to the first tooth presented by Dr. Rippier.

Dr. Atkinson: I only had a superficial examination of it, but I think Dr. Rippier has given us the only rational explanation of the condition of things. That the crown of the tooth pressing against the second molar was the cause of absorption of the anterior roots as well as the burrowing into the posterior root.

Dr. Mirick: Would you sacrifice the second molar?

Dr. Atkinson: I don't think I would. If I had known the condition I might have. I never saw a wisdom tooth where it was necessary to remove the second molar to get at it. It is only lack of instruments and a knowledge of the anatomy of the part that would lead any one to blunder on that.

Dr. Mirick: I am satisfied, in certain cases, he would sliver the ramus of the jaw a little.

Dr. Atkinson: Take a forcep and go at it. I have taken them out when they were completely covered with bone. In one of my earliest operations I remember distinctly of having to cut through the bony sac to get out the tooth. I succeeded in getting it out, but it was not pleasant either to the patient or myself.

Dr. Fry: There was a very interesting case at the clinic the other day. I should like to hear from Dr. Chapman the circumstances that attended it. I would like him to describe it.

Dr. Chapman: I will try to describe it. The patient was presented to me on Monday of last week with what I diagnosed to be a cystic tumor of the superior maxillary, proceeding from an old abscess. I left her for the clinic on Saturday, at which time I hoped Dr. Atkinson and other gentlemen would be present. At the time of my first examination I recognized a parchment-like feeling. There was a little difference of opinion among the gentlemen. I believe I was the only one who believed the antrum was not involved. I don't think it is. I injected with salt and water and got nothing through the nares or nostril. That was pretty positive.

Dr. Atkinson: What is the appearance of the substance —honey like?

Dr. Chapman: I got nothing of that character.

Dr. Atkinson: Was it hard?

Dr. Chapman: No. I got a dark colored fluid with some "cheesey" admixture. It was not offensive. If any of the gentlemen will come to my office at six o'clock, to-morrow evening, she will be there. She is very willing to be examined; she is a poor girl and I made the suggestion if she would allow me to use her in that way, it would reduce my price of treatment.

Dr. Atkinson: I would like to be allowed to make a remark or two. Many times we have tumors that encroach upon the territory of the antrum that do not involve the antral cavity proper, by reason of forcing in the lining membrane of the antrum, forming a sac, in which the growth is held; it shows the diagnostic ability of the chairman when he gives us proof that is a test for a diseased antrum, or as to its being involved when you inject it and get no flow into the nostril or back into the throat.

Dr. Chapman: We entered the sac through the socket of the palatine root of the first molar tooth.

Dr. Mirick: That would take it pretty direct into it.

Dr. Chapman: I believe by going direct through the center of the palatine root of the first molar you could strike the antrum.

Dr. Atkinson: I have seen many palatine roots of molar teeth that were entirely through the cancellous portion of the socket, and were like little nodules within antral cavity with the blood vessels and nerves running along the membrane penetrating it. In certain cases, if you extract the cuspid tooth you may enter a little projection of the antrum. I can generally tell from the person's external appearance where the antrum would be. The point I wanted to commend was the common sense way that the President went to work to determine whether it was there or not. I am only speaking from general principles, of course. I have not seen the case. There are a great many interesting cases that arise here that the physicians are utterly unable to do anything with. I have a case now, commenced last Summer, that has been said to be cured three times, and the cavity was opened inside of the cavity of the mouth into the antrum as supposed. It was into the antral territory but no discharge from the nose, and none when I made the operation. The case is cured, and the only thing that we are waiting for is the filling in of new material. The operation was such as I always make in those cases, simply burring out of the bone to healthy tissue, and as I have said many times, reducing an old sore to a clean wound.

Dr. Chapman: What would be the treatment for this case of mine?

Dr. Atkinson: I should simply wash out with warm salt and water until it came out clean, take out what did not belong there, and after that wet completely all over the sides of it, either by injection or a wad of cotton saturated with chloride of zinc, which I use where I wish union by first intention. It makes a bond of union that is the best I know of.

Dr. Mirick: This case of Dr. Chapman's recalls to mind a similar case. A young lady who came from New Orleans, just before the war, who had been in the hands of several physicians who had treated her for enlargement that was throwing out the cheek. She had been sent North to be put under treatment of some celebrated surgeon. She came to Brooklyn, and her physician recommended her to me. I examined the case, and I thought it was pretty serious. It looked to me a great deal as Dr. Chapman's. In examining closely, the bicuspid appeared to have been extracted. The gum looked healthy. I examined around the gum and I made a little opening here, which gradually wormed itself into the gum for about an inch and then struck something which I had no difficulty in diagnosing as the root of the tooth. By working at it I got underneath it and drew out the end of the root. It was the root of one of the bicuspids. In a fortnight, as Dr. Chapman says, there was a perceptible improvement in the protuberance. She returned South, and I did nothing further with it. After the war closed the young lady came to see me, with her face in an entirely normal condition; nothing was done except taking out that little root; and to my mind instead of working downward it appeared to work upward, causing this trouble. It was further up than I should expect to find a bicuspid root. Her friends came to me before they went away and wanted my bill, expecting I should charge $500 or $1,000, but I charged them fifty cents for extracting the root, and have been mad at myself ever since.

Dr. Chapman : This is the fourth cystic tumor that has been in my hands. The first I have not a very clear recollection of. The second was a small one which I cut into and stopped and plugged with cotton saturated with iodine. Another was a very large one. In opening it I saturated the parts with iodine and sulphuric acid, and it gave the patient a great deal of trouble which he did not have before I commenced to work at him. It is gradually decreasing, but there was an opening in the mouth and there is a discharge.

Dr. Atkinson : There is dead bone there, and as soon as it is dissolved away or removed artificially it will get well. There is a necrosed condition there that keeps up the discharge.

Dr. Chapman : Is there any risk in leaving it as it is ?

Dr. Atkinson : Nothing but the waste of time.

Dr. Farrar : I have now under treatment a case.

Dr Chapman : Do you find the antrum penetrated ?

Dr. Farrar : The chamber is not involved, but I can take a probe and push the lining membrane up into it. The outer plate of the superior maxillary along against the bicuspid teeth is decalcified, so bulged that it is forcing the cheek out, and as I press upon it it springs in as the bottom of a tin pan might be sprung in. It feels like cartilage, elastic. Is not rigid, and has a metallic feel. I am treating it from the outside of the gum, and to prevent the shell from remaining bulged out I am gradually forcing it in. To-day I sprung it in about a quarter of an inch. I propose to give anæsthetics and heroically force the shell in at one sitting. If we do not do that, the sac will probably fill up and the deformity remain through life.

Dr. Chapman : Don't you expect that outer plate will be dissolved away now ?

Dr. Farrar : No ; but I think it is becoming more and more decalcified. I look now for new formation and filling in of the space. It is about the size of a pigeon's egg. It is an interesting case, because it originated in a blind abscess and was seven years forming, never breaking out. I told the patient three years ago to treat it but she thought she would not. Up to a year ago it was nothing noticeable in size. I had a blind abscess in my own mouth that was five years burrowing in that way. About three or four months ago it was the size of a half filbert, and I experimented on myself. I tried the most radical means of treatment, and cured it in two or three weeks.

### Artificial Dentures.

Dr. Scott: For cheap dentistry give me celluloid.

Dr. Chapm n : Do you find it permanent?

Dr. Scott: Permanent, yes, sir; I will show you plates that have been in use six years and are good yet.

Dr. Andrews: Do you not find any trouble from the secretions of the mouth?

Dr. Scott: No; it will not soften.

Dr. Walker: Do you find any trouble from warping?

Dr. Scott: It will not warp if it is properly made. I made in July 37, August 40, September 37, October 40, November 34, December 37, January 43. I have not made a failure so far. "Never had to make one over." There is not one that I know of that is broken. I don't claim to have the best patients in the city. I have as good as the rest. For cheap work, celluloid will give better satisfaction to the public than anything else. For permanency I think there is nothing equal to gold.

Dr. Chapman: Do you use a dry press or steam?

Dr. Scott: Steam.

Dr. Mirick: You don't have any that need repairs?

Dr. Scott: Oh, yes! It fails the same as gold or rubber plates fail. I have had three this last week. Everything breaks, and celluloid for cheap work is better than rubber. The great trouble is that you make two or three sets, and you fail and you drop it. You don't keep on. Look at the work on rubber. It is the easiest thing to use. You can't make a mistake if you understand it.

Dr. Fry: I got acquainted with a dentist some time ago, and he says, "Do you use celluloid?" I replied, "No, I don't, although there are now very good men trying it; but when plates come back I don't like the looks of them." Said he, "It is the best stuff you ever saw." Pulling out a set of teeth, he says, "Look at them. Don't you think they are splendid?" It was a vile thing.

Dr. Andrews: I find there is considerable difference in the oral secretions in different individuals. For some mouths the material is softened. As a rule, I have found a strong pressure in every case essential to make a union.

Dr. Fishbough: I have used celluloid the last six years. I have made some failures, that is to say, there have been two or three special sets of celluloid that have come back to me that were blackened, grown black in the mouth by wearing. I cannot refer that change to any cause very definitely; but I do find there is an advantage; that it improves celluloid if—before you commence to screw down the flask—you allow your fire to go out, to allow the process of cooling to be set up. It seems to condense it; closes the pores and prevents that absorption of the fluids of the mouth that is liable to take place if you complete your operation while the piece is very hot. I think every one, by a moment's reflection, will see the common sense of that, and I believe many of the cases of failures have been owing to the application of too high a degree of temperature. A heat above 280° I think tends to destroy. I think it is better and safer to use a moderate heat.

Adjourned. —*Johnstons' Dental Miscellany.*

